# Innovative training networks: a new way of collaboration-propped PhD training

**DOI:** 10.1007/s00430-019-00647-0

**Published:** 2019-12-07

**Authors:** Ina Meuskens, Seamus Higson, Dirk Linke

**Affiliations:** 1 Section for Genetics and Evolutionary Biology, Department of Biosciences, University of Oslo, P.O.Box 1066, Blindern, 0316 Oslo, Norway; 2 University of Chichester, Bishop Otter Campus, College Lane, Chichester, West Sussex PO19 6PE UK

In recent years, collaborations among scientists, research groups and universities have gained more and more emphasis. With increasing complexity of experimental designs, single groups are oftentimes not able to provide all necessary facilities and experimental knowledge themselves. This development is the reason why scientists as well as policy makers bank on collaborative environments within all areas and levels of research. Among many other funding agencies, the European Union as one of the major scientific funding bodies in Europe climbed on this bandwagon. As part of the Horizon 2020 initiative, the Marie Skłodowska-Curie actions offer innovative training networks (ITNs). The main purpose is to implement collaborative research networks early on in training of the next generations of researchers during their PhDs. This special issue is the result of one of those ITNs named ViBrANT. The ‘Viral and BacteRial Adhesin Network Training’ focuses on viral and bacterial adhesion and combines expertise from academic researchers and companies from different fields of science, including basic and applied biomedical research as well as diagnostics. This special issue will give a broad overview of the network and about the current knowledge and techniques used by the members of ViBrANT.

It is hard to find a proper definition of what really constitutes a collaboration. The standard dictionary definition of the word says that collaboration is described as a group of people working towards the same goal [1]. This only superficially describes our common understanding of research collaborations: all scientists work towards the goal of gaining knowledge. Katz et al. have come up with a more appropriate definition of research collaborations, formulating criteria for what the word collaboration entails within science [2]. According to them, everyone who was involved in the project, whose name appears on the proposal and who was responsible for at least one major finding or experiment is part of a collaboration. Parties who were involved in (at least) one key step of the endeavor or the project proposal are also considered collaborators [2]. It is commonly believed that collaborations according to this definition are something positive and strengthen the scientific output of a researcher or research group. Collaborations have positive effects on different levels. The first level is the combination of different expertise and knowledge that generally functions as an internal quality control [3]. Furthermore, collaborations aid in the distribution of knowledge—within the community, knowledge is seen as a public good [4]. Third, collaborations strengthen scientific social networks and thereby enhance productivity as scientific knowledge is embedded in social structures within a research community [2]. But all of this comes at a cost. Finding and establishing a functioning collaboration is time consuming and allocating the credit for achievements can oftentimes lead to problems and conflicts [4]. Being able to rely on already established collaborations and networks is thus favorable. This is especially true for early stage researchers, on PhD level and early postgraduate level.

Many funding agencies now aid in lowering the cost of establishing collaborations by funding international, specialized groups of researchers. One of these initiatives are ITNs funded by the EU as part of the Marie Skłodowska-Curie actions within Horizon 2020 [5]. The idea behind this action is to fund PhD positions within a community of international research groups. Within this network, academic researchers and companies working on related topics come together to write a proposal for an ITN. Funding then includes the salary cost for a number of PhD students in the network, but also consumables costs, and costs for maintaining the network (travel, administration, etc.). In 2018, 123 ITNs were funded that way. With 1650 proposals submitted, this comes down to a funding rate of 7.4% [6].

One of the proposed ITNs funded since 2017 is ViBrANT, to which this special issue is dedicated. ViBrANT is a network consisting of twelve research groups, both from academic institutions and from SMEs (small and medium-sized enterprises), that all focus on research in the field of viral and bacterial adhesion [7]. Adhesion to the host is one of the initial steps during infectious processes. Infectious diseases pose a major threat to our health system, especially with antibiotic resistance in bacteria on the rise [8]. ViBrANT as a network combines basic and translational research with the aim of developing new diagnostic tools to tackle these issues. The network aspires to combine knowledge from different fields and enables both the research groups as well as the individual PhD students to work interdisciplinary and internationally [5]. Within an ITN, PhD students receive research training as well as experience in working outside their usual environment or comfort zone. This way they also learn useful ‘soft’ skills for the future and become equipped with an entire network of collaborators working on related topics [7].

Taken together, ITNs facilitate collaboration for early stage researchers, since a network is already pre-established once they start their studies. Coming together in an ITN is beneficial for the academic research groups, as they receive funding for their projects and access to complementary methods and expertise, and for the industry partners, as they get better access to expert knowledge and human resources. At the same time, it is highly beneficial for the early stage researchers, who receive excellent training, experience in working collaboratively in large, interdisciplinary teams, and a doctoral level research degree; in addition, they get access to an established network of academic and industry contacts in their field of interest. It is hard to imagine a better start to a scientific researcher career.

In the case of ViBrANT, fifteen PhD students coming from nine different countries started their work in research laboratories in six different countries in 2018. This special issue gives an overview of what the scientific topics are within ViBrANT. In addition, it includes opinion articles on researcher mobility and other topics related to ITNs that are related to biomedical research.
